# Appendicular Skeletal Muscle Mass and Sarcopenia as Prognostic Markers in Amyotrophic Lateral Sclerosis

**DOI:** 10.1002/jcsm.70378

**Published:** 2026-09-10

**Authors:** María Teresa Zarco‐Martín, María Carmen Andreo‐López, Miriam Soraya Yagui‐Beltrán, María Luisa Fernández‐Soto

**Affiliations:** ^1^ Endocrinology and Nutrition Unit San Cecilio University Hospital Granada Spain; ^2^ Foundation for Health Research in Eastern Andalusia – Alejandro Otero (FIBAO) Granada Spain; ^3^ Granada Biosanitary Research Institute (Ibs. Granada) Granada Spain; ^4^ Department of Medicine University of Granada Granada Spain

**Keywords:** amyotrophic lateral sclerosis, handgrip strength, muscle mass, sarcopenia, survival

## Abstract

**Background:**

Amyotrophic lateral sclerosis (ALS) is characterized by progressive muscle wasting and functional decline. Prognostic assessment in clinical practice relies largely on functional scales and traditional anthropometric measures. The prognostic relevance of muscle mass, muscle strength and sarcopenia in ALS remains incompletely defined. This study evaluated the prognostic value of muscle mass, muscle function and sarcopenia in relation to mortality in patients with ALS.

**Methods:**

A prospective observational cohort study was conducted at the ALS Multidisciplinary Unit (UMELA), San Cecilio University Hospital in Granada. Sarcopenia was defined according to the European Working Group of Sarcopenia in Older People 2 (EWGSOP2) and malnutrition using the Global Leadership Initiative on Malnutrition (GLIM) criteria. Muscle status was assessed using bioelectrical impedance vector analysis (BIVA)‐derived parameters, muscle ultrasound, handgrip strength (HGS) and physical performance (SPPB). Differences between survivors and nonsurvivors were analysed and logistic regression explored potential predictors of mortality. Receiver operating characteristic (ROC) curves were constructed for significant predictors. Survival was analysed using the Kaplan–Meier and Cox proportional hazards regression.

**Results:**

A total of 42 patients with ALS were included (mean age 66.1 ± 10.3 years, and 15 (36%) were female). During follow‐up (mean survival 21.1 ± 10.5 months), 45% died. Survivors showed higher appendicular skeletal muscle mass index (ASMI) (6.94 ± 0.91 vs. 5.80 ± 0.98 kg/m^2^, *p* = 0.001), body cellular mass index (BCMI) (8.92 ± 1.59 vs. 7.64 ± 1.87 kg/m^2^, *p* = 0.034) and HGS (25.45 ± 11.11 vs. 17.67 ± 8.83 kg, *p* = 0.032), while BMI did not differ (*p* = 0.540). Sarcopenia was more prevalent in nonsurvivors (27.8% vs. 4.8%, *p* = 0.047). In multivariable analysis, ASMI remained associated with mortality after adjustment for age, onset type and ALSFRS‐R (OR 0.33, 95% CI 0.11–0.81, *p* = 0.026), but this association was not statistically significant in the fully adjusted model including disease duration (*p* = 0.093). ASMI showed the highest discriminative ability (AUC 0.81), compared to BCMI (AUC 0.70) and HGS (AUC 0.71). ROC‐derived cut‐offs (ASMI 6.74 kg/m^2^; BCMI 7.2 kg/m^2^; HGS 21 kg) stratified survival. Higher ASMI (HR 7.53, *p* = 0.008), BCMI (HR 4.18, *p* = 0.005) and HGS (HR 4.02, *p* = 0.019) were associated with longer survival, whereas sarcopenia increased mortality risk (HR 15.53, *p* < 0.001).

**Conclusions:**

Beyond BMI, skeletal muscle mass—particularly ASMI—was associated with survival in ALS. Sarcopenia identifies a subgroup of patients at particularly high risk of death. Sarcopenia assessment may improve prognostic stratification and support clinical management in ALS.

## 1 Introduction

Amyotrophic lateral sclerosis (ALS) is a progressive neurodegenerative disorder characterized by marked heterogeneity in disease progression and survival [1]. Despite advances in multidisciplinary care, prognosis remains highly variable with survival ranging from months to several years [2]. Established prognostic factors, including age at onset, site of symptom onset, respiratory involvement and nutritional and functional status assessed by the ALS Functional Rating Scale–Revised (ALSFRS‐R), only partially explain this variability [3, 4, 5, 6].

Muscle wasting is a core feature of ALS and plays a central role in functional decline, nutritional impairment and mortality [7, 8]. Progressive motor neuron loss leads to denervation, rapid skeletal muscle atrophy and loss of strength. These processes are further exacerbated by hypermetabolism, reduced dietary intake, systemic inflammation and the development of malnutrition [9, 10, 11]. Accumulating evidence suggests that muscle loss should not be considered merely a consequence of disease progression but as a relevant body composition and metabolic determinant of functional decline and survival in ALS [12].

Previous studies have demonstrated that reduced muscle mass is consistently associated with shorter survival and faster disease progression in ALS [12, 13, 14]. Similarly, higher body mass index (BMI) at diagnosis and weight stability during follow‐up have been linked to improved outcomes, whereas weight loss predicts poorer prognosis [15]. However, BMI lacks sensitivity to distinguish between fat and lean tissue compartments and may fail to detect clinically relevant muscle wasting [16].

Indeed, several studies have shown that nutritional status in ALS is complex and not always reflected by conventional anthropometric indicators. In ALS, neurogenic muscle loss may develop early and precede overt malnutrition according to Global Leadership Initiative on Malnutrition (GLIM) criteria. Muscle depletion may remain undetected when relying solely on nutritional diagnoses [17].

Beyond muscle mass, muscle strength and function are also determinants of disease progression and prognosis in ALS. Handgrip strength (HGS) is strongly correlated with functional capacity and with specific domains of the ALSFRS‐R, related to motor function [18]. Declines in muscle strength translate directly into loss of independence in activities of daily living and are associated with reduced survival [8].

In recent years, several tools have been developed to assess muscle mass and function, including bioelectrical impedance vector analysis (BIVA), muscle ultrasound, HGS and physical performance tests [19]. These approaches provide complementary information on muscle quantity, quality and functional capacity, allowing a more comprehensive evaluation of muscle health than traditional anthropometric measures. However, despite their feasibility in routine clinical practice, integrated assessment of these muscle‐related parameters has not yet been systematically incorporated into ALS care. Consequently, although previous studies support the prognostic relevance of muscle loss in ALS, the clinical utility of an integrated and feasible morphofunctional assessment combining muscle mass, muscle strength, and sarcopenia has not been sufficiently explored.

Therefore, this study evaluated the prognostic value of an integrated muscle assessment, including muscle mass, muscle strength and sarcopenia, in relation to mortality in patients with ALS.

## 2 Methods

### 2.1 Study Design and Population

An observational cohort study including 42 adults diagnosed with ALS who attended a nutrition consultation in the multidisciplinary team of ALS (UMELA) at San Cecilio University Hospital in Granada was conducted between March 2023 and May 2025. People with neurodegenerative diseases other than ALS were excluded. All patients with ALS were diagnosed based on El Escorial criteria [20].

The study was approved by the Ethics Committee of Granada (Spain) following the principles of the World Medical Association Declaration of Helsinki. The project was reviewed and approved by the Research Ethics Committee of Granada on February 25, 2022 (Project ID: 1770‐N‐21).

### 2.2 Clinical Assessment

Clinical variables included disease duration, defined as the time (months) from symptom onset to baseline evaluation, site of onset (bulbar/spinal). Baseline forced vital capacity (FVC, %) and percutaneous radiologic gastrostomy (PRG) during follow‐up, up to the censoring date, were collected from clinical records.

Functional status was assessed using ALSFRS‐R score, a validated 12‐item scale evaluating bulbar, motor and respiratory function, with scores ranging from 0 to 48, where higher scores indicate better functional status [21].

Malnutrition was diagnosed according to the GLIM criteria, which require the presence of at least one phenotypic criterion (weight loss, low BMI or reduced muscle mass) and one etiologic criterion (reduced food intake or disease burden/inflammation). In this study, reduced muscle mass was assessed using appendicular skeletal muscle index (ASMI). ASMI was calculated divided by height squared (m^2^). Cut‐off points used to define low muscle mass were < 7.0 kg/m^2^ for men and < 6 kg/m^2^ for women, according to the GLIM criteria [22].

Sarcopenia was defined as low muscle strength (dynapenia) and low muscle mass (myopenia) according to the European Working Group on Sarcopenia in Older People 2 (EWGSOP2). Dynapenia criteria were assessed by HGS (males < 27 kg and females < 16 kg). Myopenia was assessed by ASMI (men < 7 kg/m^2^, women < 5.5 kg/m^2^) [23].

Survival time was defined as the time (months) from symptom onset to death or last follow‐up. In addition, for survival analyses, time‐to‐event was calculated from the date of the baseline nutritional assessment to death or censoring (May 2025).

### 2.3 Body Composition Assessment, Muscle Strength and Function

#### 2.3.1 Anthropometric Measurements

Habitual weight (kg) in the last 6–12 months was reported by the patients. Actual weight (kg) was assessed using a scale (SECA, Birmingham, UK). Height (m) was obtained using a stadiometer (SECA, Birmingham, UK). Weight loss (habitual weight‐actual weight/habitual weight × 100) and BMI (actual weight/height × height [kg/m^2^]) were calculated.

#### 2.3.2 Bioimpedance Vector Analysis (BIVA)

Phase angle (PA) and body composition analyses were obtained using Nutrilab, a 50‐kHz phase‐sensitive impedance analyser (Akern, Florence, Italy). The PA was expressed in degrees as arctan (Xc/R) × (180°/p). An individual standardized PA value (SPA) was determined by adjusting it by sex and age. Data obtained using BIVA for body composition were categorized as fat‐free mass (FFM/height, kg/m), fat mass (FM/height, kg/m), total body water (TBW/height, kg/m), extracellular water (% ECW of TBW), body cellular mass (BCM, kg), body cellular mass index (BCMI, kg/m^2^), skeletal muscle mass index (SMI, kg/m2) and appendicular skeletal muscle mass (ASMM, kg), obtained from predictive equations. The equation proposed by Kyle et al. was used for participants aged 18–65 years, whereas the equation proposed by Sergi et al. was used for participants aged > 65 years [24, 25]. Normality values from Nutrilab were used [26]. ASMI (kg/m^2^) was calculated as ASMM divided by height squared (m^2^).

BIVA was conducted following established guidelines to ensure accuracy and reproducibility. Participants were positioned in a supine position with their limbs slightly apart to prevent skin contact, which could interfere with the electrical impedance measurement and to ensure stability. A 5‐min rest period in the supine position was ensured prior to measurements to minimize the impact of fluid shifts caused by changes in posture. The BIVA device was calibrated daily according to the manufacturer's instructions, and the accuracy of the BIVA measurements was verified using a precision circuit provided by the manufacturer.

#### 2.3.3 Nutritional Ultrasound

Muscle ultrasonography was conducted on the quadriceps rectus femoris (QRF) of the lower extremity using a 10‐ to 12‐MHz probe and a multifrequency linear matrix (Mindray Z60, Madrid, Spain). This examination was performed on all subjects while they were in a supine position. The assessment was carried out without applying compression, positioned at the lower third from the superior pole of the patella and the anterior superior iliac spine. Measurements included assessing anteroposterior muscle thickness, circumference and cross‐sectional area. The ultrasonography procedure was performed by an individual previously trained in this technique. The probe was positioned perpendicular to the longitudinal and transverse axes in the QRF to measure parameters such as the rectus femoris cross‐sectional area (RF‐CSA), RF‐axis (*Y*‐axis) and L‐SAT (subcutaneous fat of the leg). The same trained individual conducted the ultrasonography, performing three measurements for each parameter, and the mean value was calculated.

For assessing adipose tissue, the midpoint between the xiphoid appendix and the navel was identified for imaging in the abdominal area. Here, measurements were taken for T‐SAT (total subcutaneous abdominal fat) and VAT (preperitoneal or visceral fat) in centimetres [27].

#### 2.3.4 Muscle Strength

Muscle strength was assessed using an adult dynamometer (Jamar handgrip dynamometry, Asimow Engineering Co., Los Angeles, CA, USA) and was measured in the dominant limb, repeated on three occasions. The highest value was used to represent HGS.

#### 2.3.5 Physical Performance

Physical performance was analysed using the Short Physical Performance Battery (SPPB) [28], which includes three domains: balance, walking speed, and getting up from and sitting down on a chair five times. Each test is scored from 0 (worst performance) to 4 (best performance). A total score is obtained which is the sum of the three tests and ranges from 0 to 12.

### 2.4 Statistical Analysis

Statistical analyses were performed using R software (version 4.1.2; RStudio, PBC, Boston, MA, USA). The distribution of continuous variables was assessed using the Shapiro–Wilk test. Continuous variables are presented as mean ± standard deviation, while categorical variables are expressed as absolute numbers and percentages.

Comparisons in clinical and body composition, muscle strength and function variables according to survival status were evaluated. Student's *t*‐test for independent samples or the Mann–Whitney *U* test was applied for continuous variables, depending on normality. Categorical variables were compared using the Chi‐square test or Fisher's exact test.

Univariate logistic regression analyses were first conducted to evaluate the association between baseline clinical, body composition, muscle strength and functional variables and mortality status at the end of follow‐up. Variables showing significant associations (*p* < 0.05) in univariate models were subsequently entered into multivariable logistic regression models to identify independent predictors of mortality. Multivariable logistic regression models were additionally performed including adjustment for established ALS prognostic factors (age, ALSFRS‐R score, disease duration and site of onset), both individually and in combined models, to assess the robustness of the association under different confounding structures.

The discriminatory capacity of variables independently associated with mortality was assessed using receiver operating characteristic (ROC) curve analysis. The area under the curve (AUC) with 95% confidence intervals was calculated, and optimal cut‐off points were determined using the Youden index.

Survival analyses were performed using the Kaplan–Meier method, and survival curves were compared between groups defined by ROC‐derived cut‐off values using the log‐rank test. The Cox proportional hazards regression models were used to estimate hazard ratios (HRs) and 95% confidence intervals for mortality. Time‐to‐event was defined as the interval between the baseline nutritional assessment (performed at study inclusion) and death or censoring at the end of follow‐up. In the Cox models, analyses were additionally adjusted for age and ALSFRS‐R score to account for baseline differences in key prognostic factors. The proportional hazards assumption was assessed both graphically and analytically.

Although disease duration was defined from symptom onset, baseline clinical and nutritional measurements were obtained at the time of the nutritional assessment. Logistic regression analyses were used as a complementary approach to survival analyses to evaluate the association between baseline variables and mortality status at the end of follow‐up, providing a cross‐sectional estimate of risk that was subsequently complemented by time‐to‐event modelling.

## 3 Results

### 3.1 Study Population Characteristics Differences According to Survival Status

A total of 42 ALS people were included, 36% were women, with a mean age of 66.1 ± 10.3 years old. Bulbar onset was present in 54% of cases. Nineteen patients (45%) died during the study period. The mean survival was 21.14 ± 10.5 months. Study population characteristics are shown in Table 1.

**TABLE 1:** Baseline clinical, body composition and muscle‐related characteristics of ALS patients according to survival.

	Total (42)	Survival (23)	Nonsurvival (19)	*p* ^a^
Age (years)	66.1 (10.3)	62 (10.3)	71.2 (8.2)	**0.004**
Sex (F/M)	15/27	8/15	7/12	0.718
Onset type (B/S)	23/19	13/10	9/10	0.554
Dis. duration (months)	20.6 (14.8)	26.7 (16.1)	16.4 (12.1)	**0.039**
BMI (kg/m^2^)	24.60 (4.60)	25.96 (5.11)	23.06 (3.75)	0.540
Weight loss (%)	5.89 (7.89)	3.54 (5.45)	9.13 (10.4)	**0.049**
PA (°)	4.71 (0.84)	4.89 (0.78)	4.56 (0.87)	0.234
BCM (kg)	22.50 (5.90)	24.28 (5.00)	19.64 (5.54)	**0.012**
BCMI (kg/m^2^)	8.33 (1.88)	8.92 (1.59)	7.64 (1.87)	**0.034**
ASMI (kg/m^2^)	6.45 (1.24)	6.94 (0.91)	5.80 (0.98)	**0.001**
ECW (%)	52.90 (5.32)	51.74 (4.62)	53.96 (5.66)	0.203
Na/K	1.27 (0.26)	1.25 (0.30)	1.31 (0.22)	0.571
FMI (kg/m^2^)	6.38 (3.41)	6.82 (3.89)	5.52 (2.52)	0.254
RF‐CSA (cm^2^)	3.69 (1.59)	3.67 (1.30)	3.59 (1.83)	0.875
RF‐Y axis (cm)	1.21 (0.38)	1.25 (0.33)	1.13 (0.36)	0.274
L‐SAT (cm)	0.73 (0.67)	1.00 (0.68)	0.42 (0.59)	**0.010**
A‐SAT (cm)	1.52 (0.88)	1.65 (1.07)	1.32 (0.59)	0.330
VAT (cm)	0.60 (0.44)	0.61 (0.32)	0.51 (0.22)	0.331
HGS (kg)	21.40 (11.10)	25.45 (11.11)	17.67 (8.83)	**0.032**
SPPB	7.19 (2.90)	7.14 (2.53)	7.06 (3.35)	0.927
ALSFRS‐R	36.70 (8.26)	39.38 (5.63)	35.17 (8.33)	0.069
Malnutrition, *n* (%)	17 (40.5)	6 (28.6)	9 (50)	0.170
Sarcopenia, *n* (%)	8 (19.0)	1 (4.8)	5 (27.8)	**0.047**

*Note:* Values in bold emphasis highlight the statistical significance.

Abbreviations: ALSFRS‐r, revised amyotrophic lateral sclerosis functional rating scale; ASMI, appendicular skeletal muscle mass index; B, bulbar onset; BMI, body mass index; BCM, body cell mass; BCMI, body cell mass index; Dis. duration, disease duration; ECW, extracellular water; F, female; FMI, fat mass index; HGS, hand grip strength; M, male; Na/K, sodium potassium index; PA, phase angle; RF‐CSA, rectus femoris cross‐sectional area; RF‐Y axis, rectus femoris thickness; S, spinal onset; SAT, subcutaneous adipose tissue (L‐, leg, A‐, abdominal); SPPB, short performance physical battery; VAT, visceral adipose tissue.

^a^ Mann–Whitney U test or *t*‐test for continuous variables; Chi^2^ or Fisher for categorical variables. Results are expressed as mean (SD) (numeric variables) or %(n) (categorical variables).

At baseline, survivors exhibited less weight loss during the previous 6 months (3.54% vs. 9.13%, *p* = 0.049) and had better muscle mass and function. Specifically, survivors presented higher BCM (24.28 ± 5.00 vs. 19.64 ± 5.54 kg, *p* = 0.012), BCMI (8.92 ± 1.59 vs. 7.64 ± 1.87 kg/m^2^, *p* = 0.034), ASMI (6.94 ± 0.91 vs. 5.80 ± 0.98 kg/m^2^, *p* = 0.001) and HGS (25.45 ± 11.11 vs. 17.67 ± 8.83 kg, *p* = 0.032). Survivors also had greater leg subcutaneous adiposity (1.00 ± 0.68 vs. 0.42 ± 0.59 cm, *p* = 0.010).

The prevalence of sarcopenia was significantly higher among nonsurvivors (27.8% vs. 4.8%, *p* = 0.047), whereas malnutrition showed a nonsignificant difference (50% vs. 28.6%, *p* = 0.170).

No significant differences were observed in BMI (*p* = 0.450), lower limb ultrasound‐derived muscle morphology (*p* = 0.875; *p* = 0.274) or global physical performance (*p* = 0.927).

### 3.2 Mortality Predictors in ALS

In univariate logistic regression analyses, higher values of BIVA‐derived muscle mass parameters—BCM, BCMI and ASMI—were significantly associated with lower odds of mortality. Specifically, BCM (OR 0.84, 95% CI 0.71–0.96; *p* = 0.022), BCMI (OR 0.64, 95% CI 0.40–0.96; *p* = 0.043) and ASMI (OR 0.29, 95% CI 0.12–0.69; *p* = 0.005) emerged as protective factors with ASMI showing the strongest association.

Regarding muscle function, higher HGS was significantly associated with lower odds of mortality (OR 0.92, 95% CI 0.84–0.99; *p* = 0.045), whereas global physical performance assessed by the SPPB was not associated with survival.

**FIGURE 1:**
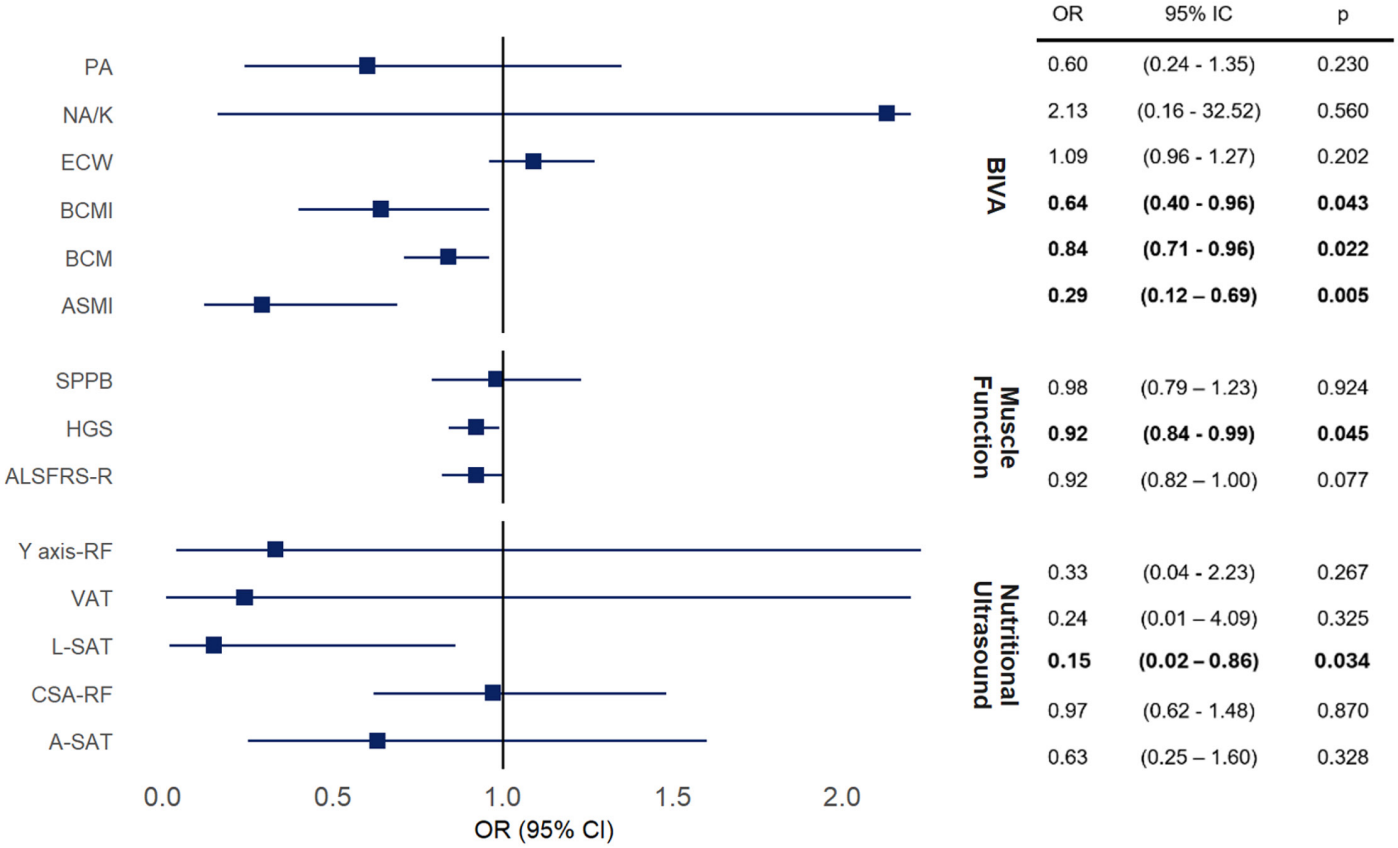
Univariate logistic regression analysis of body composition and muscle function predictors of mortality in ALS. ALSFRS‐r: revised amyotrophic lateral sclerosis functional rating scale; ASMI: appendicular skeletal muscle mass index; BCM: body cell mass; BCMI: body cell mass index; ECW: extracellular water; HGS: Hand grip strength; M: male; Na/K: sodium potassium index; PA: Phase angle; RF‐CSA: rectus femoris cross‐sectional area; RF‐Y axis: rectus femoris thickness; SAT: subcutaneous adipose tissue (L‐: leg, A‐: abdominal); SPPB: Short Performance Physical Battery; VAT: visceral adipose tissue.

No significant associations were observed for cellular quality markers (PhA [OR 0.60, 95% CI 0.24–1.35; *p* = 0.230], Na/K ratio [OR 2.13, 95% CI 0.16–32.52; *p* = 0.560]), hydration status (ECW [OR 1.09, 95% CI 0.96–1.27; *p* = 0.202]) or most ultrasound‐derived muscle morphology parameters, including CSA‐RF (OR 0.97, 95% CI 0.62–1.48; *p* = 0.870) and muscle thickness (OR 0.33, 95% CI 0.04–2.23; *p* = 0.267). Among regional adiposity measures, only greater L‐SAT was associated with lower odds of mortality (OR 0.15, 95% CI 0.02–0.86; *p* = 0.034) (Figure 1). Higher FVC showed a borderline association with lower odds of mortality (OR 0.969, 95% CI 0.938–1.000; *p* = 0.050).

In domain‐based multivariable analysis, ASMI was associated with risk of mortality (OR 0.38, 95% CI 0.13–0.89, *p* = 0.040), whereas muscle strength (HGS) was not independently associated with mortality (OR 0.97, 95% CI 0.87–1.06, *p* = 0.477).

To account for potential confounding, additional multivariable logistic regression analyses were performed adjusting for established ALS prognostic factors (Table 2). Lower ASMI remained associated with mortality after adjustment for ALSFRS‐R score (OR 0.31, 95% CI 0.11–0.70, *p* = 0.011), age (OR 0.35, 95% CI 0.12–0.82, *p* = 0.026), disease duration (OR 0.37, 95% CI 0.13–0.86, *p* = 0.034) and site of onset (OR 0.28, 95% CI 0.10–0.62, *p* = 0.005). In combined models adjusting for ALSFRS‐R and age (OR 0.33, 95% CI 0.11–0.81, *p* = 0.026) and for ALSFRS‐R, age and site of onset (OR 0.33, 95% CI 0.11–0.81, *p* = 0.026), the association remained consistent. In the fully adjusted model including all covariates simultaneously, the association showed a similar effect size but did not reach statistical significance (OR 0.42, 95% CI 0.13–1.10, *p* = 0.093).

**TABLE 2:** Association between ASMI and mortality across adjusted logistic regression models.

Model	OR	95% CI	*p*
Adjusted for ALSFRS‐R	0.31	0.11–0.70	0.011
Adjusted for age	0.35	0.12–0.82	0.026
Adjusted for onset type	0.28	0.10–0.62	0.005
Adjusted for disease duration	0.37	0.13–0.86	0.034
Adjusted for age and ALSFRS‐R	0.33	0.11–0.81	0.026
Adjusted for age, ALSFRS‐R and onset type	0.33	0.11–0.81	0.026
Adjusted for age, ALSFRS‐R, onset type and disease duration	0.42	0.13–1.10	0.093

Abbreviations: ALSFRS‐r, revised amyotrophic lateral sclerosis functional rating scale; ASMI, appendicular skeletal muscle mass index.

### 3.3 Prognostic Value of Muscle‐Related Parameters

**FIGURE 2:**
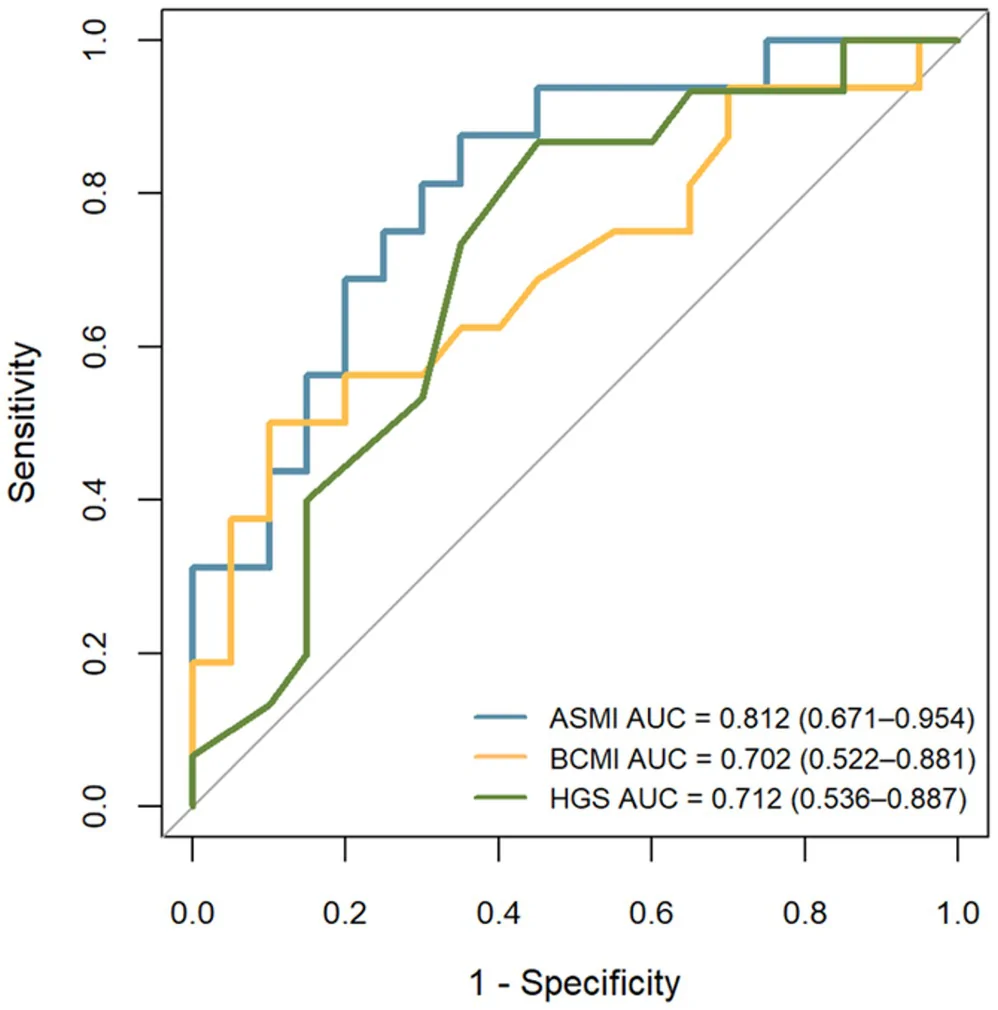
ROC curves of muscle‐related parameters for mortality prediction in ALS. ASMI: appendicular skeletal muscle mass index; AUC: area under the curve; BCMI: body cell mass index; HGS: handgrip strength.

ROC curve analysis identified optimal cut‐off points for BCMI, ASMI and HGS for predicting survival in ALS. BCMI showed an AUC of 0.70 (95% CI: 0.52–0.88) with a cut‐off of 7.20 (sensitivity 0.50, specificity 0.90); ASMI had an AUC of 0.81 (95% CI: 0.67–0.95) with a cut‐off of 6.74 (sensitivity 0.88, specificity 0.65), and HGS had an AUC of 0.71 (95% CI: 0.54–0.89) with a cut‐off of 21.00 (sensitivity 0.80, specificity 0.65) (Figure 2).

**FIGURE 3:**
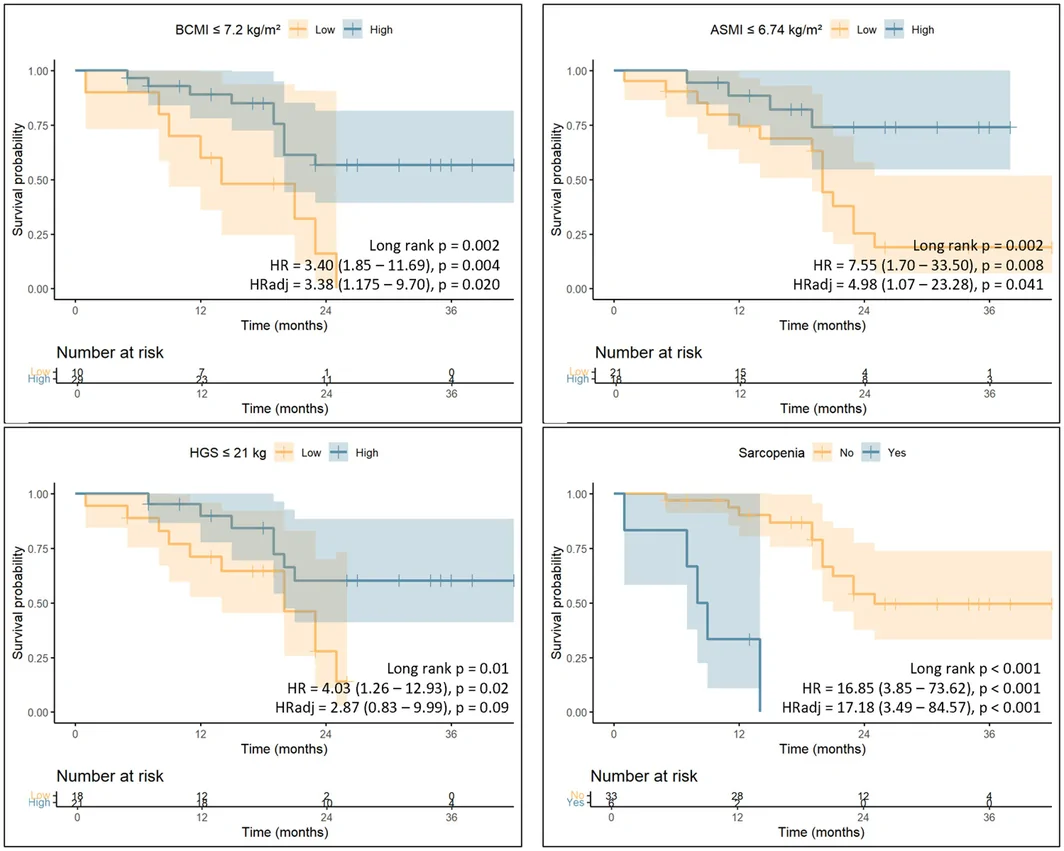
Kaplan–Meier survival curves according to muscle mass, muscle strength, and sarcopenia status. ASMI: appendicular skeletal muscle mass index; BCMI: body cell mass index; HGS: Hand grip strength; HR: hazard ratio.

Using ROC‐derived cut‐off values, the Kaplan–Meier survival analyses showed that higher muscle mass strength and no sarcopenia were associated with longer survival. In the Cox proportional hazards models adjusted for age and ALSFRS‐R, lower muscle mass and strength parameters were associated with increased mortality risk. Patients with BCMI below the cut‐off had a significantly higher risk of death (HR 3.38, 95% CI 1.17–9.71, *p* = 0.024). Similarly, low ASMI was associated with mortality (HR 4.98, 95% CI 1.07–23.28, *p* = 0.041). Although lower HGS showed a trend towards increased mortality risk, this association did not reach statistical significance after adjustment (HR 2.88, 95% CI 0.83–9.99, *p* = 0.096). In contrast, sarcopenia was strongly associated with increased mortality risk (HR 17.19, 95% CI 3.49–84.57, *p* < 0.001) (Figure 3).

## 4 Discussion

In this study, skeletal muscle mass—particularly ASMI—was associated with survival in patients with ALS. Survivors presented higher muscle mass indices and greater HGS at baseline, whereas sarcopenia was more prevalent among non‐survivors. No significant differences were observed between groups in BMI or the prevalence of malnutrition. These findings suggest that muscle mass‐related parameters may provide relevant prognostic information in ALS.

To our knowledge, this is the first study in ALS that has integrated the evaluation of muscle mass indices, muscle strength and sarcopenia in relation to mortality. Our findings suggest that muscle‐related parameters assessment may improve mortality risk stratification and support the potential value of incorporating muscle measures into clinical monitoring and management strategies.

Survivors exhibited significantly higher baseline values of BIVA‐derived muscle mass parameters, including BCM, BCMI and ASMI. Among these, ASMI was associated with mortality after adjustment for age, onset type and ALSFRS‐R. This association was also observed across several adjusted models, although it was not statistically significant in the fully adjusted model including all covariates simultaneously. These findings suggest that muscle mass may provide prognostic information complementary to conventional clinical markers, although this observation should be considered exploratory. In addition, ASMI showed the highest discriminative performance in ROC analyses and showed potential utility for survival stratification in the Kaplan–Meier analysis. These findings are consistent with previous studies showing that reduced muscle mass, assessed by computed tomography (CT), dual‐energy X‐ray absorptiometry (DXA) or ultrasound‐based techniques, is associated with shorter survival [13, 29, 30] and faster disease progression in ALS [13, 29, 30]. However, CT and DXA are limited by cost and, in the case of CT, involve radiation exposure, restricting their use in routine clinical practice. In contrast, BIVA‐derived ASMI represents a safe, inexpensive and feasible for repeated assessment in clinical settings.

Longitudinal studies have demonstrated that progressive loss of FFM and lean body mass predicts accelerated functional decline and increased mortality risk [12, 29]. Together, these observations suggest that muscle wasting may reflect clinically relevant disease burden and metabolic impairment beyond global disease severity, supporting its role as a clinically relevant prognostic marker in ALS. Our findings are also consistent with emerging biomarkers, such as the sarcopenia index (ratio of creatinine to cystatin C), which has been shown to have promising prognostic value in ALS [31].

Although BMI has consistently been reported as a prognostic marker in ALS [32, 33], our findings underscore the limitations of BMI as an isolated marker of body composition. In our cohort, BMI did not differ between survivors and nonsurvivors despite significant differences in muscle mass and strength. Similarly, the prevalence of malnutrition at baseline was comparable between survival groups. While previous studies have reported an association between malnutrition and poorer survival in ALS [34], the lack of association in our cohort may reflect the relatively preserved BMI and the early stage of nutritional deterioration in many patients. Indeed, muscle depletion may precede overt malnutrition in ALS and is likely related to the neurogenic processes underlying muscle loss [7, 17, 35].

Notably, several patients presented preserved BMI alongside low BCMI, reflecting reduced cellularity and altered body composition. These observations illustrate the inability of BMI to distinguish between FM and lean tissue. This limitation is particularly relevant in ALS, where muscle wasting may occur without overt weight loss. Consistent with previous body composition studies, loss of FFM and lean body mass, rather than BMI per se, appears to be more closely associated with progression and mortality [12].

HGS was significantly lower in nonsurvivors and was associated with mortality in univariate analyses, as well as with reduced survival in the Kaplan–Meier models. Nevertheless, this association did not remain statistically significant after adjustment for age and ALSFRS‐R, suggesting that its prognostic value may be partly mediated by global disease severity. Although the prognostic role of HGS in ALS has been less frequently explored, our findings are consistent with previous studies showing that reduced muscle strength, assessed by manual muscle testing (MMT) scores and Medical Research Council (MRC) scores, is associated with poorer outcomes in ALS [36, 37].

However, when muscle strength was evaluated in a domain‐based multivariable model that accounted for skeletal muscle mass, HGS did not remain independently associated with mortality. According to EWGSOP2, muscle strength should be interpreted in relation to the amount of available muscle mass, as this relationship reflects muscle quality and functional efficiency rather than quantity alone [23]. In ALS, muscle strength may be influenced not only by muscle mass depletion but also by denervation patterns, motor unit dysfunction and compensatory reinnervation mechanisms [38]. Consequently, preserved strength may transiently coexist with ongoing muscle wasting. This may explain why ASMI, as a measure of skeletal muscle reserve, remained associated with mortality, whereas HGS lost significance after adjustment for muscle mass.

While ASMI captures global appendicular muscle mass, HGS assesses force generation in a single upper limb muscle group and may not fully reflect whole‐body muscle reserve in ALS. These findings support the importance of comprehensive assessment of muscle mass and function rather than relying on isolated strength measures for prognostic evaluation in ALS.

Sarcopenia was more prevalent among nonsurvivors and was associated with an increased risk of mortality. Among the variables evaluated, sarcopenia showed one of the strongest associations with survival, suggesting its potential value as an integrated marker of disease severity in ALS. These findings are consistent with the concept of ALS as a model of disease‐related sarcopenia, characterized by accelerated muscle loss occurring independently of chronological age and primarily driven by neurogenic mechanisms [39]. However, the EWGSOP2 criteria were developed primarily for age‐related sarcopenia and may not fully capture the specific characteristics of neurogenic muscle loss in ALS. In this context, the diagnosis of sarcopenia in ALS should be interpreted cautiously, as reduced muscle strength and mass may also reflect denervation and motor neuron loss rather than age‐related sarcopenia alone.

ALS and sarcopenia share overlapping pathophysiological pathways, including progressive motor unit loss, muscle fibre atrophy, mitochondrial dysfunction, oxidative stress and chronic low‐grade inflammation, which contribute to impaired muscle regeneration and functional decline [8, 39, 40]. In ALS, these mechanisms may be further aggravated by hypermetabolism, reduced intake, malnutrition and cachexia and may contribute to more pronounced muscle depletion and poorer clinical outcomes [10, 34, 41]. Recent studies on frailty and cachexia in ALS suggest that sarcopenia represents a central component of a broader vulnerability phenotype and is associated with mortality [42].

Recent evidence suggests a link between muscle‐related phenotypes and neuroaxonal biomarkers in ALS. Neurofilament light chain (NfL), a well‐established biomarker of neuroaxonal injury, has consistently been associated with faster disease progression and poorer survival [43]. In addition, Zulueta A. et al. reported an association between skeletal muscle mass and NfL levels, supporting a potential interaction between neurodegeneration and muscle loss in ALS [7]. It is plausible that patients with higher NfL levels, reflecting greater motor neuron injury, may be accompanied by more rapid denervation‐related muscle atrophy, leading to earlier development of sarcopenia and poorer clinical outcomes. In this context, NfL and muscle‐related parameters may provide complementary prognostic information, reflecting different dimensions of ALS pathology, namely, neuroaxonal injury and its downstream consequences on muscle reserve. The association between sarcopenia and mortality observed in our cohort further supports the relevance of muscle‐related phenotypes as markers of disease severity and prognosis in ALS.

From a clinical perspective, sarcopenia may capture the cumulative burden of reduced muscle quantity, impaired muscle function and systemic metabolic stress more comprehensively than isolated parameters [39]. The association between sarcopenia and mortality observed in our cohort supports the potential relevance of incorporating sarcopenia screening and muscle mass assessment into routine ALS evaluation from the time of diagnosis. Importantly, muscle depletion may occur before substantial weight loss or overt malnutrition becomes clinically evident, suggesting that traditional nutritional markers alone may underestimate early nutritional and muscular deterioration.

In this context, simple and feasible tools such as handgrip dynamometry and BIVA‐derived muscle mass indices, particularly ASMI, may facilitate early identification of high‐risk patients and improve longitudinal monitoring and risk stratification. Given the absence of effective pharmacological therapies to reverse muscle loss in ALS, early recognition of low muscle mass or sarcopenia may also have therapeutic implications, supporting closer nutritional surveillance, earlier optimization of protein and energy intake and individualized rehabilitative strategies aimed at preserving muscle function and physical performance.

This study has several strengths, including the comprehensive evaluation of muscle‐related parameters through a multimodal approach, integrating body composition, muscle strength and sarcopenia assessment, as well as the analysis of survival outcomes in a real‐world clinical setting. The use of standardized and clinically feasible tools enhances the translational relevance of these findings.

However, several limitations should be acknowledged. The relatively small sample size and limited number of events (*n* = 19) reduce the statistical power and precision of multivariate estimates, particularly in models including several covariates. Therefore, these findings should be interpreted as exploratory and hypothesis‐generating rather than confirmatory. In addition, the single‐centre design may limit the generalizability of the results. Nevertheless, the clinical characteristics of our cohort are generally comparable to those reported in previous European ALS studies, particularly regarding age at disease onset [34]. The proportion of bulbar onset cases in our cohort was somewhat higher than that reported in several population‐based series, which may reflect referral patterns to our tertiary multidisciplinary ALS clinic and should be considered when extrapolating our findings to the broader ALS population. Survival in ALS is highly heterogeneous and may vary according to demographic characteristics, disease phenotype, progression rate and methodological differences in survival definitions across studies. Although the mean survival observed in our cohort was lower than that reported in some large population‐based registries [43], it remains within the range described in previous ALS cohorts [34, 37]. Furthermore, newer phenotypic classifications such as the OPM (onset, propagation and motoneuron dysfunction) system were not systematically collected and therefore could not be retrospectively applied to our cohort [44]. Future studies incorporating more detailed phenotypic stratification may help clarify whether the prognostic value of muscle mass and sarcopenia differs across ALS phenotypes. Information on PEG placement was available; however, the type and intensity of nutritional interventions, including the use of high‐calorie or high‐protein diets, were not systematically recorded. Similarly, the use of disease‐modifying treatments such as riluzole or edaravone was not systematically available. Baseline FVC was significantly lower in nonsurvivors than in survivors; however, its association with mortality was only borderline in univariate analysis, and it was not further evaluated in multivariable models because of the limited number of events. These factors may influence nutritional status, muscle preservation, disease progression and survival, and their absence from the analyses may therefore have contributed to residual confounding. Finally, muscle mass was estimated using predictive equations which are widely used for estimating skeletal muscle mass. However, they were not specifically developed or validated in ALS populations, which may limit the accuracy of muscle mass estimation in patients with neurodegenerative disease and altered body composition. Owing to the observational design, no conclusions regarding causality between muscle‐related parameters and survival outcomes can be drawn, and external validation in larger multicentre cohorts is warranted.

## 5 Conclusion

ASMI appears to be a relevant prognostic factor in patients with ALS, while sarcopenia identifies a subgroup at higher risk of mortality. These findings may support the role of muscle‐related parameters as clinically useful markers for risk stratification. Importantly, ASMI and sarcopenia may also have value as enrichment or stratification tools in future ALS clinical trials by helping to identify patients with distinct prognostic profiles. However, these observations should be considered exploratory and require validation in larger prospective cohorts.

## Funding

This study was funded by the Sociedad Española de Nutrición Clínica y Metabolismo (SENPE) through the 2024 Award for the Best Clinical Nutrition Research Project by Young Investigators, granted to the first author. In addition, M.T.Z‐M is funded by a postdoctoral fellowship from Granada Biosanitary Research Institute (Ibs. Granada) (74‐2022).

## Ethics Statement

The present study was carried out in accordance with the latest version of the Declaration of Helsinki. The study was approved by the Ethics Committee of San Cecilio University Hospital, Granada, Spain (1770‐N‐21).

## Conflicts of Interest

The authors declare no conflicts of interest.

## Data Availability

The data presented in this study will be made available upon request to the corresponding author.
